# The role of neuromuscular function in sleep apnea pathogenesis and management: A consensus of experts

**DOI:** 10.3389/frsle.2023.1087196

**Published:** 2023-01-30

**Authors:** Reena Mehra, Richard Schwab, R. John Kimoff, Neomi Shah, Daniel J. Gottlieb, Sanjay R. Patel, Najib T. Ayas, Patrick Hanly, Geoff Sheean, Atul Malhotra

**Affiliations:** ^1^Sleep Disorders Center, Neurologic Institute, Respiratory Institute, Lerner Research Institute, Cleveland Clinic, Cleveland, OH, United States; ^2^Division of Sleep Medicine, Department of Medicine, University of Pennsylvania, Philadelphia, PA, United States; ^3^Respiratory Division, McGill University Health Centre, Montreal, QC, Canada; ^4^Respiratory Epidemiology Clinical Research Unit and Meakins-Christie Laboratories, McGill University, Montreal, QC, Canada; ^5^Icahn School of Medicine at Mount Sinai, New York, NY, United States; ^6^Boston VA Medical Center and Brigham, Women's Hospital, Boston, MA, United States; ^7^Center for Sleep and Cardiovascular Outcomes Research, University of Pittsburgh, Pittsburgh, PA, United States; ^8^Department of Medicine, University of British Columbia, Vancouver, BC, Canada; ^9^Cumming School of Medicine, Sleep Centre, Foothills Medical Centre, Hotchkiss Brain Institute and Department of Medicine, University of Calgary, Calgary, AB, Canada; ^10^Department of Regenerative Medicine, University of California, San Diego, San Diego, CA, United States

**Keywords:** obstructive sleep apnea, neuromuscular function, polysomnography, pathogenesis, upper airway

## Abstract

**Study objectives:**

Although the importance of upper airway assessment in the consideration of obstructive sleep apnea (OSA) is recognized, there are current limitations in our approach to assessment.

**Methods:**

We convened a group of experts in upper airway neuromuscular physiology and anatomy, sleep apnea endophenotypes, novel therapeutics and sleep epidemiology to summarize existing literature and delineate future opportunities to utilize and incorporate innovative and less invasive techniques focused on upper airway neuromuscular physiology to assess and manage OSA.

**Results:**

In OSA, genioglossus electromyogram (EMG) activity is reduced during sleep onset with higher levels observed during wakefulness compared to controls. Surface EMG recordings are limited due to distance from the actual muscle and while needle EMG offers more direct assessment, this approach is more invasive. Novel alternatives overcoming these limitations to assess upper airway neuromuscular physiology in OSA may therefore prove beneficial. Specifically, such an approach would facilitate identification of upstream prognostic biomarkers of OSA clinical trajectory and offer more informative mechanistic data. Novel approaches to neuromuscular assessment in OSA would enhance phenotyping to predict better tolerance to positive airway pressure therapy and set the stage to target neuromuscular function and upper airway anatomy. A quantifiable and repeatable neuromuscular physiologic metric has potential to facilitate a precision medicine strategy and personalize treatment, including measuring treatment response to neurophysiologic-focused interventions including hypoglossal nerve stimulation (HGNS), myofunctional therapy and neuromuscular electrical stimulation. A key area for future investigation is whether observed neuromuscular changes can identify patients at future risk of OSA, facilitating early intervention or prevention strategies.

**Conclusions:**

Overall, recognizing the critical contributions of abnormalities of upper airway neuromuscular function to the pathophysiology of OSA, it may be important to find accurate and reproducible neurophysiological assessments to address existing knowledge gaps in OSA assessment and management.

## Introduction

Obstructive sleep apnea (OSA) is a common disease with major neurocognitive and cardiometabolic sequelae (Jenkinson et al., 1999; Gottlieb and Punjabi, 2020). OSA is estimated to affect up to 1 billion people worldwide with the majority of disease remaining undiagnosed and untreated (Benjafield et al., 2019). Diagnostic strategies for OSA have evolved over time from gold standard polysomnography (PSG), which is often regarded as cumbersome and expensive to home sleep apnea testing (HSAT) which provides adequate diagnostic accuracy albeit imperfect (Kuna et al., 2011; Rosen et al., 2012). Despite ongoing efforts, the mechanistic insights which are derived from sleep diagnostics are currently minimal, suggesting a need for more advanced approaches.

OSA pathogenesis is recognized to involve a complex interplay of soft tissue/craniofacial structure, neuromuscular dysfunction, arousal threshold and control of breathing abnormalities (Younes, 2003, 2004; Jordan et al., 2014). Many patients develop OSA primarily due to anatomical abnormalities including increased size of the upper airway soft tissue structures (tongue, soft palate, lateral pharyngeal walls, tonsils, etc.) or small bony craniofacial structures (e.g., retrognathia, micrognathia), while in other patients abnormalities in upper airway dilator muscle function, control of breathing, or arousal threshold play a more prominent role (Edwards et al., 2014). Some patients have combinations of abnormalities that contribute to OSA (Edwards et al., 2016). These underlying mechanisms or endotypes may be important to guide personalized therapeutic interventions and may facilitate mitigation of apnea-related complications if adequately addressed (Malhotra et al., 2020).

At present, the gold standard for OSA diagnosis remains PSG, but this test is relatively cumbersome and expensive and not readily available for large portions of the population (Mulgrew et al., 2007; Kuna et al., 2011). HSAT is increasingly accepted as an alternative approach given less burden and expense. However, both PSG and HSAT generally fail to discriminate among the various mechanistic subtypes of OSA that might inform the personalized therapeutic decisions. Moreover, neither HSAT nor PSG is sufficiently accessible within existing medical insurer coverage paradigms to allow repeated administration as a means of serial testing e.g., in response to therapy. In addition, neither test can be administered in the clinic to provide straightforward assessment at the point of care. While deriving endophenotypes from PSG data is an area of active research (Sands et al., 2018a), the increasing reliance on HSATs, which provide less data than PSG, may hinder the widespread implementation of precision medicine approaches that are dependent on PSG data. Moreover, there is no currently available diagnostic tool that is predictive of the future development of OSA in at-risk individuals or progression of OSA in those with mild disease.

The ideal diagnostic test for OSA would provide reliable and reproducible information in terms of identifying patients who will benefit from interventions. It would be inexpensive, easy to use and non-invasive to allow widespread use of the technology and have high sensitivity and specificity for OSA. It would also be readily accessible in the clinic to allow straightforward assessment at the point-of-care (Montesi et al., 2012). The approach would ideally integrate characterization of mechanistic aspects of OSA. In addition, the ability to make serial assessments of OSA severity and provide information regarding underlying mechanisms longitudinally with ease and minimal burden to patients would provide an opportunity to use the technique to guide therapy and to gauge therapeutic response, e.g., to titrate medications or neuromuscular training/stimulation. As a point of emphasis, a technique to assess OSA severity may be useful diagnostically whereas another technique to allow determination of OSA mechanisms may provide therapeutic guidance. In theory, one method could inform both diagnostics and therapeutics although these two distinct goals need to be considered when validating and implementing new methods. Taking into consideration patient preference is also critical to ensure patient receptivity with implications on engagement in the diagnostic process as well as ultimately treatment adherence as indicated (Table 1).

**Table 1:** Ideal obstructive sleep apnea-specific diagnostic challenges characteristics of potential solutions.

**Obstructive sleep apnea-specific diagnostic challenges to address**	**Attributes**	**Benefits**
Neurophysiologic-focused endophenotypes	Provides mechanistic data	Facilitates precision medicine approach
Large, global disease burden	Sensitive for disease	Screening and diagnostic utility
Expense of diagnostics	Specific for disease	Avoid unnecessary additional testing
Apnea hypopnea index imperfect	Correlates with disease severity	Quantify disease burden, prioritize therapy
Currently a one-size-fits all approach	Treatment responsive	Measure and monitor treatment response
Polysomnography not readily available and cumbersome	Point-of-Care	Efficiency, accessibility, scalability
Expense of diagnostics	Inexpensive	Accessibility, scalability, cost-savings
Polysomnography is cumbersome and inconvenient	Non-invasive	Patient satisfaction and ease of use
Manual scoring is labor-intensive	Easily measured	Does not require major expertise to assess
Current standard is single night testing	Quantitative metric	Serial assessment
Patient-centered decision making	Consideration of patient receptivity and preference	Enhanced adherence and patient satisfaction

Given the impact of OSA and limitations of current diagnostic and management approaches, we convened a panel with expertise inclusive of upper airway neuromuscular physiology and anatomy, sleep apnea endophenotypes, novel therapeutics and sleep epidemiology to discuss the potential of neuromuscular function measurement in the assessment of OSA. Our objectives included summarizing the current literature and identifying opportunities for future research and product development. The session was sponsored by Powell Mansfield, Inc., but the sponsor had no role in the academic content or in drafting the manuscript. Mansfield, Inc. is a parent company with subsidiaries of OSA diagnostic companies including Powell Mansfield.

## Unmet needs in OSA evaluation and diagnostics

The current practice of sleep medicine relies on diagnosis of established OSA; however, there may be antecedent abnormalities which could identify people destined to develop OSA in the future or who may have transient risk of OSA, for example in pregnancy and during procedural anesthesia and sedation. Although at present, we do not have robust preventative strategies for OSA beyond the general health measures of diet and exercise (Awad et al., 2012; Carneiro-Barrera et al., 2022), a technique that allowed early identification of a high-risk population could facilitate development and testing of novel preventive approaches, and also facilitate targeting of intensive lifestyle intervention to those most likely to benefit. One situation where prognostication would have immediate value would be in the perioperative setting (Ayas et al., 2018). Patients without OSA may manifest OSA postoperatively in the context of benzodiazepine and opioid administration (Robinson et al., 1985, 1987; Ayas et al., 2018). Such patients are likely at risk for OSA pre-operatively, but are only identified postoperatively in the face of perturbations. Although questionnaire-based approaches have been shown to have value in OSA screening in the post-operative setting (Chung et al., 2008), approaches with higher accuracy and integrating direct neuromuscular upper airway physiology may improve efficiency of OSA diagnostic paradigms in the post-operative setting with the potential to improve outcomes.

Several practical challenges exist with the current diagnostic paradigm in OSA such as cost, efficiency, accessibility, and scalability. The gold standard PSG is a time-consuming and costly test with limited availability for large portions of the population, involves multiple clinical steps and is performed under atypical sleeping conditions in a sleep lab. The emergence of HSAT has clear value as it has enhanced accessibility and facilitated the diagnosis of OSA without the need for in-laboratory PSG for many patients. However, HSAT has its own limitations, including lack of a sleep assessment for many devices (Malhotra and Ayas, 2020). Although clinical experience suggests utility in the context of patients with major comorbidities, data validating its use in this context are relatively weak. HSAT often involves Level 3 testing without electroencephalogram, thereby limiting accuracy of sleep apnea severity assessment as monitoring time rather than sleep time is used in the calculation of the apnea hypopnea index (AHI) and electroencephalographic (EEG) arousals are not considered with the scoring of hypopnea events (Berry et al., 2022). This situation results in increased likelihood of false negative results, particularly in those with more mild or subtle sleep-disordered breathing. The multi-faceted aspects of the ideal diagnostic test for OSA have been described, and would provide informative mechanistic data with ease and little burden while generating sensitive and specific measures of OSA that track with disease severity and treatment responsiveness (Montesi et al., 2012).

In addition to the practical challenges, current diagnostic approaches frequently fail to provide mechanistic insight into the pathophysiology of OSA in an individual patient. Several investigators have attempted to improve mechanistic insights gleaned from PSG using sophisticated signal processing algorithms (Orr et al., 2018; Sands et al., 2018a), although the clinical utility of these methods has yet to be validated. While these efforts are ongoing with respect to both HSAT and PSG, given the importance of upper airway neuromuscular function to OSA pathogenesis (discussed below), direct measurement of neuromuscular function offers a promising approach that may provide valuable mechanistic information to the more routinely available HSAT and PSG metrics. While traditional methods of neuromuscular assessment are invasive and impractical for routine clinical use, newer non-invasive techniques may be more widely applicable. That said, in this context, an important nuance that bears mention is the difference between OSA endotype (which provides mechanistic information about the mechanisms underlying OSA) versus phenotypes (clinical expression of disease ranging from asymptomatic to severe sleepiness).

## Unmet needs in OSA therapeutics and management

The current gold standard for OSA treatment is continuous positive airway pressure (CPAP). CPAP is highly efficacious for control of OSA in most patients and may have transformative benefits for patients who use it consistently, as it can produce major improvement in symptoms and associated improvement in blood pressure, snoring and perhaps cardiovascular risk (Pepperell et al., 2002; Marin et al., 2005). Despite its efficacy, CPAP is not always well tolerated, thus limiting its effectiveness (Schwab et al., 2013). Efforts to improve CPAP adherence are ongoing using intensive education and support, modern technology allowing patient engagement, innovative sleep scoring of the diagnostic PSG to identify patients who are more likely to use CPAP and efforts to improve the patient experience *via* mask fitting, facilitating nasal patency (Hoy et al., 1999; Malhotra et al., 2018; Benjafield et al., 2021; Younes et al., 2022), etc. Enhanced OSA phenotyping represents an opportunity area that may allow for ability to predict better tolerance or intolerance to CPAP therapy. However, intolerance or suboptimal adherence to CPAP are likely to continue to limit its effectiveness in many patients.

Therefore, the field of sleep medicine is moving beyond the one-size-fits all approach to treatment (Mazzotti et al., 2019) with CPAP therapy. Alternative therapies exist that target either upper airway anatomy (e.g., mandibular repositioning devices and surgical modification of the upper airway) or neuromuscular function (e.g., hypoglossal nerve stimulation (HGNS), myofunctional therapy, neuromuscular electrical stimulation) (Guimaraes et al., 2009; Maghsoudipour et al., 2021; Nokes et al., 2022a,b). These alternative therapies are individually less efficacious than CPAP, however, underscoring the need for accurate and reliable endotypic and phenotypic measures to identify patients likely to benefit from these different interventions. Specific endotypes such as arousal threshold and high loop gain have been derived as endo-phenotyping using polysomnography (PUP) with clinically meaningful applications (Sands et al., 2018b). Neuromuscular physiologic mechanistic data independent of (or complementing) diagnostic HSAT or PSG data may be valuable in identifying specific OSA endotypes that would guide a more personalized approach to disease management. Integration of point of care already described endotypic measures such as breath-hold responses for loop gain (Messineo et al., 2018) or blood sample testing [e.g., bicarbonate levels (Zou et al., 2023)] combined with novel approaches to ascertain upper airway neuromuscular function has potential to considerably advance and optimize current clinical approaches. If a quantifiable and repeatable metric could be developed, it could facilitate a precision medicine strategy and personalize treatment, including measuring treatment response.

As one example, HGNS is FDA approved and while there are data to suggest individual characteristics which predict response to therapy, there is a need for further refinement to identify *a priori* patients who are likely to respond to (or fail) the intervention (Bachour et al., 2021). Some data suggest that age, gender, body mass index may have predictive value (Heiser et al., 2019; Pascoe et al., 2022), but a rigorous evaluation of neuromuscular function may offer biologically relevant insights to predict treatment response. The use of drug-induced sleep endoscopy (DISE) is typically used to stratify the candidates for HGNS based upon type of anatomical collapse, but this approach is invasive, and expensive and may have issues around reproducibility of collapse type (intra and inter-observer on repeated measures). Thus, there is an opportunity to investigate and develop alternative methods to risk stratify patients prior to HGNS to improve treatment response and clinical outcomes.

An increasing body of evidence indicates that myofunctional therapy targeting the upper airway muscles during wakefulness may improve OSA severity and symptoms in mild-to-moderate OSA (de Felicio et al., 2018; Hsu et al., 2020; O'Connor-Reina et al., 2020; Rueda et al., 2020; Carrasco-Llatas et al., 2021). Standardized approaches to assess the evaluation of the stomatognathic system have been validated using the orofacial myofunctional evaluation (OMES) protocol which has facilitated ability to diagnose orofacial myofunctional disorders (de Felicio et al., 2010). Approaches involving soft palate elevation, tongue and buccofacial exercises (de Felicio et al., 2018) applied several times per week have led to significant improvements in AHI and Epworth Sleepiness Scale scores in OSA patients compared with controls (Hsu et al., 2020; Rueda et al., 2020). However, the factors– including neuromuscular physiology– determining which patients are most likely to respond, as well as optimal approaches to therapy, remain unclear and require further investigation.

Another area of interest is the use of neuromuscular electrical stimulation to activate and train upper airway muscles (Guimaraes et al., 2009; Nokes et al., 2022a,b). While randomized studies have not yet been reported, studies with a daytime oropharyngeal stimulation intervention have shown improvement in snoring and mild sleep apnea with this intervention (Guimaraes et al., 2009; Nokes et al., 2022a,b). However, at present, we lack an ability to predict a priori who may respond to this intervention, perhaps suggesting that an assessment of neuromuscular function may have value in distinguishing responders and non-responders. Of note, prior physiological studies in OSA have shown increased strength of the genioglossus on a tongue protrusion task, but with reduced endurance compared to controls (Eckert et al., 2011). Neuromuscular electrical stimulation has been shown to improve endurance in physiological studies, although research is ongoing in this area (Nokes et al., 2022b). *In vitro* studies have similarly demonstrated increased fatigability of human genioglossus samples from non-obese OSA patients compared with controls or CPAP-treated OSA patients (Carrera et al., 2004). These findings suggest a potentially modifiable aspect of upper airway neuromuscular function, although further data are clearly required.

Thus, at present, our ability to identify which patients are likely to respond to HGNS, myofunctional therapy or neuromuscular electrical stimulation remains unclear. A reliable technique utilized during sleep or wakefulness assessing neuromuscular function may therefore be helpful in guiding appropriate therapy. In theory, some patients who are amenable to these interventions could be identified and prioritized for daytime neuromuscular therapy, whereas patients unlikely to respond to daytime measures may be directed to alternative interventions.

## Neuromuscular function in OSA

Considerable research has been performed regarding upper airway neuromuscular function in OSA (Kimoff et al., 2001; Nguyen et al., 2005; Kimoff, 2007). Several conclusions have been suggested, although debate is ongoing in this context. First, some evidence of sensory impairment has been observed in the upper airway although the pathophysiological relevance of these findings is unclear (Wallace et al., 2022). Second, regarding motor function, the genioglossus has been shown to have increased strength but reduced endurance in OSA compared to controls (Eckert et al., 2011). Third, a negative pressure reflex has been described whereby the genioglossus and other muscles in the upper airway will activate in response to a negative (sub-atmospheric, collapsing) pressure stimulus (Mathew, 1984). The negative pressure reflex has been shown to be increased not decreased in OSA compared to controls (Berry et al., 2003). Fourth, histological studies have been performed in biopsy specimens from patients with OSA (Series et al., 1995). Series et al. showed a highly trained upper airway muscle in OSA based on fiber type distributions (red, type 1, slow twitch fibers) compared to non-OSA controls (Series et al., 1995). Fifth, some authors have suggested that the mechanical output of the upper airway muscles may be impaired even with adequate neural stimulation (Patil et al., 2004; Jordan et al., 2007). So-called “neuromechanical uncoupling” has been postulated although direct evidence has been more controversial. In aggregate the data suggest that upper airway neuromuscular function may be important for some but not all OSA patients.

The heterogeneity of the underlying pathophysiologic contributions to OSA has been recognized such that four main endophenotypic traits have been identified, i.e., (1) pharyngeal anatomy/collapsibility, (2) ventilatory control system gain (loop gain), (3) the ability of the upper airway to dilate/stiffen in response to an increase in ventilatory drive, and (4) arousal threshold (Jordan et al., 2014). Intersection with upper airway neuromuscular physiology can be influenced by and can influence these OSA pathophysiologic traits directly and/or indirectly. It has been speculated that upper airway sensory dysfunction in OSA may reflect an OSA-related neuropathic process which may also impact motor innervation (Saboisky et al., 2012a; Wallace et al., 2022). Indeed, several lines of evidence point to upper airway muscle denervation-reinnervation in OSA. Histopathologic studies of OSA upper airway tissue demonstrate fiber type grouping (Lindman and Stal, 2002), myocyte expression of neurotrophic factors (Boyd et al., 2004) as well as pathologic axonal and Schwann cell changes (Shah et al., 2018). In addition, conventional genioglossus intra-muscular electrode recordings by several groups have demonstrated classical denervation potentials (Svanborg, 2005; Saboisky et al., 2012a) in some OSA patients vs. controls. Vibration injury– perhaps in combination with hypoxia– has been suggested to contribute to the observed neuromuscular abnormalities in OSA (Saboisky et al., 2012b). While these changes clearly have the potential to impact upper airway motor function, their prevalence and pathophysiologic significance remain poorly understood. Further study will be required to determine if the observed neuromuscular changes can identify patients at future risk of OSA, facilitating early intervention or prevention strategies.

## Assessment of neuromuscular function in OSA

A reliable technique for direct measurement of neuromuscular function has the potential to fill some of the unmet needs in OSA diagnosis and management. However, the sleep medicine field has faced multiple challenges with regards to assessing upper airway neuromuscular physiology. Sensory testing is notoriously subjective and cannot easily be performed during sleep (Nguyen et al., 2005). Upper airway muscle biopsies are invasive with attendant risk of bleeding and infection. Intramuscular EMG can be painful and also has modest risk (e.g., bleeding, infection), and cannot easily be applied to multiple locations within a muscle without repeated instrumentation (Malhotra et al., 2000a). Cost and accessibility have also limited the ability to assess neuromuscular function of the upper airway muscles. For example, intramuscular EMG requires costly specialized equipment that is not readily available. In addition, diagnostic interpretation of intramuscular electromyogram (EMG) recordings requires specialized training and expertise in the field of neurophysiology, making testing hard to standardize between patients and within patients on repeated measurements between physicians. Thus, efforts to optimize accuracy of these measurements are ongoing. Novel approaches to implement point-of-care neuromuscular physiological assessments of the upper airway in OSA are worth pursuing. For example, a protocol-based challenge involving sustained negative pressure through a mask or device to evoke neuromuscular output, i.e., an awake neuromuscular stress test can be considered.

The genioglossus is the best studied of the upper airway dilator muscles, although there are 23 pairs of muscles that are thought to be important in upper airway patency (Horner et al., 1991; Mezzanotte et al., 1992; Horner, 2000). The genioglossus is accessible and thus potentially the easiest muscle to study. In addition, it is a large muscle thought to have important mechanical influences on pharyngeal airway patency (Innes et al., 1995). The genioglossus is a phasic muscle (bursts with inspiration) and is state dependent (changes firing pattern from wakefulness to sleep) (Horner, 1996). At present the gold standard to assess genioglossus activity is intramuscular electromyography using needle or fine wire electrodes (Malhotra et al., 2000b), although it is best used for low-level short contractions and tends to be sensitive to the movement of the electrodes, and as noted above has the disadvantage of being invasive by nature (O'Connor et al., 2007). A simplified, non-invasive technique is therefore desirable for several reasons: to reduce patient discomfort; to reduce cost; to allow assessment of multiple muscles, providing a more thorough evaluation of upper airway neuromuscular function rather than relying on the assumption that genioglossus activity is representative of all the muscles in the upper airway; and to facilitate repeated measurements over time.

Recently, a new electrodiagnostic technology called transmembranous EMG has been developed that addresses some of these challenges (Menon et al., 2022). The technology uses a novel sensor and probe that allows a diagnostic assessment of the neuromuscular function of upper airway muscles without the need for intramuscular electrodes. The transmembranous EMG signal allows measurement of muscle activity but also provides diagnostic information regarding underlying pathologies e.g., myopathy, neuropathy, etc. Due to its minimally invasive nature, multiple muscles can be examined at many different locations without undue burden. Although muscle physiology assessments may have value if they allow the assessment of neuromuscular upper airway pathophysiology to become more accessible, there is need for foundational reproducibility studies and enhanced understanding of how collection of these daytime measures relate to sleep-specific OSA pathophysiology. Further validation of the method will be required and how best to integrate this approach clinically and in the research setting is still to be determined. Of note, additional potential value in use of the transmembranous EMG is to facilitate the assessment of active perception and tongue movement, not only passive, neuromuscular measurements (Guilleminault et al., 2019). One potential advantage of a neuromuscular assessment during wakefulness would be related to feasibility; however, its ability to predict behavior/function during sleep will need further investigation.

## Future directions

Although the expert panel agreed on the importance of upper airway assessment in the consideration of OSA, there was also consensus about the need for further validation of newer techniques such as transmembranous EMG and demonstration of its utility in both research and clinical settings. Hence, there was discussion regarding potential future use cases for innovative and less invasive techniques spanning across OSA mechanistic, diagnostic, usability, prognostic, and therapeutic guidance domains (Table 2).

**Table 2:** Consensus regarding opportunities for innovations in upper airway neuromuscular physiologic assessments in the diagnosis and management of obstructive sleep apnea.

**Domain**	**Statements**
Mechanistic	• Investigation of whether noninvasive upper airway neurophysiologic measurements and related endophenotypes also provide insight into disease mechanisms. Moreover, the addition of mechanistic insight may help in terms of patient management. • Clarifying prevalence of denervation or myopathic changes among OSA vs. non-OSA patients including the extent that these changes relate to symptoms, disease severity and disease progression. • Understanding how neurophysiologic measurements can be used to assess responses to targeted therapy. • Evaluating neurophysiologic differences across age, sex, race and other important OSA subgroups.
Diagnostic	• In theory, if a point-of-care inexpensive test were available in a clinical setting, a screen for OSA could be conducted on a large scale. A point of care test would have value if highly sensitive (help to rule out disease) or highly specific (help to rule in disease). One approach could be used to enhance existing screening methods such as questionnaires (STOP-BANG, MAP, Berlin) assuming it provided additional predictive value (Netzer et al., 1999; Chung et al., 2008).
Usability	• Ease of use of the neuromuscular test at the point of care and cross-disciplinary use from clinicians of different specialty type without specialized training to conduct the test and obtain a diagnostic interpretation is a key consideration.
Prognostic	• At present we do not have robust methods to prevent OSA beyond diet and exercise for weight management. However, patients may have high risk of developing future OSA based on neurogenic abnormalities. The appropriate treatment of these patients is unclear, but clinical studies could certainly target these high risk patients to prevent incident OSA and mitigate downstream consequences (symptom-based and adverse clinical outcomes) of untreated OSA if effective interventions were available.
Therapeutic	• Given that a number of interventions are now available to treat snoring and OSA targeting upper airway muscle physiology, a useful approach may be to follow these patients serially over time. In theory, hypoglossal nerve stimulation or daytime neuromuscular stimulation or training could be titrated to daytime neuromuscular pathophysiological assessments which might allow optimization of therapy. In addition, such findings may motivate patient behavior e.g., in the context of weight loss patients may be motivated to pursue and sustain diet and exercise interventions if they received supportive feedback regarding the efficacy of their interventions. • Therapeutic guidance could also be provided to patients averse to CPAP if pathophysiological data were helpful in determining the optimal non-CPAP intervention. • In pharmacotherapeutic approaches for OSA, optimal dosing of medications could potentially be facilitated by measurement of upper airway neuromuscular function assuming the mechanism of action of these medications is *via* enhanced hypoglossal motor output. For example, in the case of atomoxetine plus oxybutynin, the serial measurement of upper airway muscle dilator activity may have therapeutic value.

OSA is now recognized to be a heterogenous disease from the perspective of underlying pathophysiological mechanisms (endotypes) as well as in terms of varying clinical expression (phenotype). The mechanisms underlying OSA include collapsible pharyngeal anatomy, dysfunction in upper airway dilator muscles, low arousal threshold, and unstable ventilatory control as well as other factors. In theory, knowledge of underlying mechanism may guide therapeutic interventions, although rigorous outcome data are lacking regarding this approach. Oxygen or acetazolamide would be predicted to improve patients with unstable control of breathing (Edwards et al., 2012), whereas hypoglossal nerve stimulation or muscle training exercise may help preferentially those patients with upper airway dilator muscle dysfunction. Regarding phenotypes, the data suggest various clusters in which some OSA patients are asymptomatic, some have comorbid insomnia and some have daytime sleepiness (Ye et al., 2014). Of note, OSA patients with hypersomnia as well as comorbid insomnia (Lechat et al., 2022) are thought to be at cardiovascular risk, suggesting that only a subset of OSA patients may experience improvements in cardiovascular risk when treated for OSA. Therefore, recognition of OSA endophenotypes is challenging the “one-size-fits-all” approach of giving nasal CPAP to all OSA patients regardless of mechanism or symptoms (Bosi et al., 2017; Malhotra et al., 2020). Furthermore, as investigated in prior work (Overby et al., 2022), there is also a role for clinical decision-making tools based upon patient interactive physiologic assessments and endophenotypic risk for more personalized approaches to OSA diagnosis and management.

Overall, recognizing the critical contributions of abnormalities of upper airway neuromuscular function to the pathophysiology of OSA (Mazzotti et al., 2019) may be important to find accurate and reproducible neurophysiological assessments to fill existing gaps not only in refinement of our understanding of underlying mechanisms, but also to aid in identification of novel endophenotypes and to enhance management efficiencies in OSA clinical pathways.

## Author contributions

All authors listed have made a substantial, direct, and intellectual contribution to the work and approved it for publication.
